# Assessment of Surgical Complications With Respect to the Surgical Indication: Proposal for a Novel Index

**DOI:** 10.3389/fsurg.2021.638057

**Published:** 2021-02-18

**Authors:** Grégoire B. Morand, Nanina Anderegg, Tobias Kleinjung, Jörg E. Bohlender, Dorothe Veraguth, Martina A. Broglie, David Holzmann, Alexander M. Huber, Christof Röösli, Michael B. Soyka

**Affiliations:** ^1^Department of Otorhinolaryngology–Head and Neck Surgery, University Hospital Zurich, Zurich, Switzerland; ^2^Faculty of Medicine, University of Zurich, Zurich, Switzerland; ^3^Institute of Social and Preventive Medicine (ISPM), University of Bern, Bern, Switzerland

**Keywords:** survey, surgeon, otolaryngologist, hearing loss, quality control, paresis

## Abstract

**Introduction:** The Clavien–Dindo classification is a broadly accepted surgical complications classification system, grading complications by the extent of therapy necessary to resolve them. A drawback of the method is that it does not consider why the patient was operated on primarily.

**Methods:** We designed a novel index based on Clavien–Dindo but with respect to the surgical indication. We surveyed an international panel of otolaryngologists who filled out a questionnaire with 32 real case-inspired scenarios. Each case was graded for the surgical complication, surgical indication, and a subjective rating whether the complication was acceptable or not.

**Results:** Seventy-seven otolaryngologists responded to the survey. Mean subjective rating and surgical complication grading for each scenario showed an inverse correlation (*r*^2^ = 0.147, *p* = 0.044). When grading the surgical complication with respect to the surgical indication, the correlation with the subjective rating increased dramatically (*r*^2^ = 0.307, *p* = 0.0022).

**Conclusion:** We describe a novel index grading surgical complications with respect to the surgical indication. In our survey, most respondents judged a complication as acceptable or not according to its grade but kept in mind the surgical indication. This subjective judgment could be quantified with our novel index.

## Introduction

A common tool to assess surgical complications is the so-called Clavien–Dindo grading system, which classifies surgical complications by the extent of the therapy that is necessary to resolve them (1). For example, a complication requiring revision in the operating theater is worse than a complication “only” requiring blood transfusion. The Clavien–Dindo classification has found widespread acceptance in the literature, and inaccurate terms such as minor or major complications are now far less often used (1). The Clavien–Dindo classification also allows to code for a sequela occurring after surgery, with the letter “d” for disability that can be added to the surgical grade of the complications if necessary. This integrates the principle of some previous surgical classification systems differentiating outcomes after surgery into three groups, namely, complication, failure to cure, and sequelae (2).

The Clavien–Dindo classification has been primarily designed for and by general and visceral surgeons (1) but is being validated and is gaining acceptance in further surgical fields such as urology (3), maxillofacial surgery (4), thoracic surgery (5), neurosurgery (6), and otolaryngology–head and neck surgery (7). The Clavien–Dindo classification is usually being adapted to better match the spectrum of surgical complications in a particular surgical field. In otolaryngology–head and neck surgery, complications such as sensorineural hearing loss, loss of smell, facial nerve palsy, or recurrent laryngeal nerve palsy would be graded quite mildly on a Clavien–Dindo classification, as they usually do not require intensive care unit treatment or endanger the life of the patient. However, these complications may be very bothersome and among the most important for surgeons, patients, and family.

Another drawback of the Clavien–Dindo classification is the fact that it does not consider the reason why the patient was operated on in the first place. In daily clinical practice, most would, however, agree that a marginal mandibular nerve palsy after a parotidectomy for a 2.5-cm cystadenolymphoma is worse than the same complication in a patient undergoing a segmental mandibulectomy and bilateral neck dissection for advanced oral cancer (8, 9). Similarly, sensorineural hearing loss after routine tympanoplasty seems worse than after resection of a large vestibular schwannoma. The large spectrum of surgical indications in otolaryngology from lifestyle surgery (e.g., esthetic rhinoplasty) to vital surgery (emergency tracheotomy, cancer surgery) through non-vital yet important interventions (tympanoplasty for chronic perforation of the tympanic membrane, medialization laryngoplasty for recurrent nerve palsy) requires the integration of the surgical indication for an accurate and fair evaluation.

We therefore aimed to establish an index for the assessment of surgical complications with respect to the surgical indication. We designed a questionnaire with a short description of 32 clinical scenarios inspired from real cases. We then surveyed a panel of international otolaryngologists from different backgrounds and experience and asked them to fill out the questionnaire. The participants graded the surgical complication and the surgical indication. Finally, a judgment if the complication, in the context of the surgical indication, is acceptable or not was made.

## Materials and Methods

As this report is a survey study with fictional clinical scenarios, no ethics approval is required according to the local ethics review board committee, *Kantonale Ethikkomission Zürich*.

### Design of Surgical Index

We first adapted the Clavien–Dindo classification by transforming it into a numerical system and integrating loss of sensory organ into the system to better match with the spectrum of complications in otolaryngology–head and neck surgery (Table 1). We then designed a new scale for surgical indications. For this scale, surgical indications were divided in seven categories, grading lifestyle surgery (e.g., esthetic rhinoplasty) as “1;” meanwhile, ultra-urgent surgery for a life-threatening condition (e.g., emergency tracheotomy for acute respiratory distress) received the highest grade (“7”) (Table 2).

**Table 1:** The modified Clavien–Dindo classification.

**Clavien–Dindo**	**Adapted**	**Complication**
I	1	Any deviation from the normal postoperative course without the need for pharmacological treatment or surgical, endoscopic, and radiological interventions
II	2	Requiring pharmacological treatment with drugs other than such allowed for grade 1 complications
IIIa	3	Requiring surgical, endoscopic, or radiological intervention in local anesthesia
IIIb	4	Requiring surgical, endoscopic, or radiological intervention in general anesthesia
IVa	5	Life-threatening complication requiring intensive care unit management with single-organ dysfunction or permanent loss of organ function
IVb	6	Life-threatening complication requiring intensive care unit management with multi-organ dysfunction
V	7	Death

**Table 2:** Grading of surgical indications.

**Grading**	**Surgical indication**
1	Lifestyle surgery, no medical indication
2	Lifestyle surgery, with medical indication
3	Disabling disease if left without surgical treatment
4	Emergency surgery >6 h <24 h
5	Lethal disease if left without surgical treatment
6	Emergency surgery within 6 h
7	Ultra-urgent surgery with direct intervention in trauma room or in intensive care unit

### Questionnaire and Surveying

We then set up a questionnaire of 32 clinical scenarios based on real-life cases from each surgical subspecialty in otolaryngology–head and neck surgery (GBM, MAB: head and neck surgery; CR, AMH, DV: otology/lateral skull base surgery and audiology; TK: salivary gland diseases; JEB: laryngology; DH, MBS: rhinology/central skull base surgery). After being designed, each question was tested, adapted if necessary, and internally validated by all co-authors of the study (Supplementary Material).

The survey was then published online *via* LimeSurvey® (V3.22.15+200505), and otorhinolaryngologic doctors worldwide were invited to participate, with a reminder electronic mailing 2 and 8 weeks after the initial electronic mailing. Participants were asked to fill out the questionnaire assessing for each clinical case scenario: the surgical indication, the surgical complication, and finally give a subjective assessment if the complication was fully acceptable (2), partially acceptable (1), and unacceptable (0).

After grading the scenarios, participants were asked to complete a series of six questions assessing the experience in otolaryngology and training grade. Participants also had the opportunity to write down any comments or concerns regarding the cases and the grading system. These comments were collected and assessed for common themes and suggestions. The time spent on each question was also recorded for each participant.

### Statistical Analysis

#### Descriptive Statistics

For each case, we calculated the mean and standard deviation for the grade of complication and surgical indication. The subjective rating is reported as the most common response (with percentage of respondents giving that response) for each case and with a mean and standard deviation of the numerical values (10) for each case. For complication, surgical indication, and subjective rating, we eliminated the two cases with the poorest agreement, that is, the highest standard deviation (10).

#### The Indication to Complication Index

We constructed a surgical index [from now on called Indication to Complication Index (ICI)] assessing surgical complication with respect to the indication. We calculated the ICI by dividing the score of surgical indication by the score of surgical complications and taking the logarithm of that value [thus, ICI = log (surgical indication/surgical complication)].

#### Performance of the Indication to Complication Index

In the first step, we checked performance of the new indicator by assessing the association between the mean (aggregated over each case) of the ICI and the mean subjective rating using Pearson coefficient of correlation for two independent continuous variables. We compared it to the corresponding correlations for the surgical complication and surgical indication.

In the second step, we compared means of ICI among the three rating groups (cases deemed to be unacceptable, partially acceptable, and fully acceptable) by a linear mixed-effect model with the outcome ICI and as explanatory variable the subjective rating group (as factor). To account for clustering, we added a random intercept for responder-ID. We used residual plots to check for normality and homoscedasticity.

In all analyses, a *p*-value lower than 0.05 was considered to indicate statistical significance. Statistical analyses were performed using SPSS® 25.0.0.1 software (IBM®, Armonk, NY, USA) and R (R core team 2020, Vienna).

## Results

### Descriptive Statistics

The survey was completed by 77 respondents, of which 45 respondents (58.4%) completed the survey fully. Of all participants, 37 (48.1%) declared to be board-certified otolaryngologists, and eight (10.4%) declared to be in training. In addition, 45 participants declared to be “in ENT business” for a median of 15 years (Q25–Q75 7–23).

Table 3 details the grading for each clinical scenario with the surgical complications graded from 1 to 7 (Table 1), the surgical indication graded from 1 to 7 (Table 2), and the subjective rating of the particular case.

**Table 3:** Descriptive statistics with distribution of grading for each clinical scenario.

**Clinical scenario**	**Complication mean (*SD*)**	**Indication mean (*SD*)**	**Subjective (%)**	**Subjective mean (*SD*)**	**Complication to Indication Index (ICI) mean (*SD*)**	**No. of respondents**
Case 1	3.6 (0.79)	2.2 (0.62)	Partially acceptable (48.1%)	1.4 (0.59)	−0.5 (0.41)	77/77
Case 2	1.4 (0.83)	1.7 (0.60)	Fully acceptable (66.2%)	1.6 (0.50)	0.2 (0.61)	74/77
Case 3	4.7 (0.77)	4.8 (1.0)	Fully acceptable (77.5%)	1.7 (0.44)	0.0 (0.23)	71/77
Case 4	4.3 (0.78)	4.8 (1.1)	Partially acceptable (62.1%)	1.3 (0.54)	0.1 (0.22)	66/77
Case 5	3.7 (0.69)	2.5 (0.98)	Fully acceptable (62.9%)	1.6 (0.55)	−0.4 (0.39)	62/77
Case 6^*^	3.9 (1.4)^*^	2.4 (0.59)	Unacceptable (59.3%)	0.54 (0.72)^*^		59/77
Case 7^*^	3.7 (1.7)^*^	4.2 (1.3)^*^	Partially acceptable (58.9%)	1.0 (0.64)		57/77
Case 8	3.9 (0.39)	2.8 (0.64)	Fully acceptable (52.7%)	1.2 (0.62)	−0.3 (0.24)	55/77
Case 9	3.9 (0.62)	4.3 (0.97)	Fully acceptable (68.5%)	1.6 (0.46)	0.1 (0.36)	53/77
Case 10	1.9 (0.34)	1.0 (0.23)	Fully acceptable (79.2%)	1.7 (0.46)	−0.6 (0.31)	53/77
Case 11	3.9 (0.41)	2.9 (0.83)	Fully acceptable (50.0%)	1.4 (0.57)	−0.3 (0.35)	52/77
Case 12^*^	4.9 (0.99)	3.4 (1.2)^*^	Partially acceptable (59.6%)	1.0 (0.63)		52/77
Case 13	4.8 (0.87)	5.9 (1.1)	Fully acceptable (53.8%)	1.4 (0.66)	0.2 (0.22)	52/77
Case 14	4.3 (1.1)	2.2 (0.65)	Unacceptable (74.5%)	0.29 (0.54)	−0.7 (0.50)	51/77
Case 15	2.1 (1.3)	2.7 (0.50)	Fully acceptable (84.0%)	1.8 (0.43)	0.4 (0.59)	50/77
Case 16	3.8 (0.97)	3.2 (0.82)	Fully acceptable (68.0%)	1.6 (0.60)	−0.1 (0.45)	50/77
Case 17	4.2 (1.2)	3.9 (1.0)	Fully acceptable (52.0%)	1.5 (0.54)	−0.0 (0.43)	50/77
Case 18	2.8 (0.65)	3.8 (1.0)	Fully acceptable (83.7%)	1.8 (0.37)	0.3 (0.42)	49/77
Case 19	3.1 (1.3)	4.3 (1.0)	Fully acceptable (57.0%)	1.9 (0.30)	0.4 (0.58)	49/77
Case 20	4.2 (1.2)	2.5 (0.58)	Partially acceptable (61.2%)	0.9 (0.62)	−0.5 (0.47)	49/77
Case 21	2.7 (1.3)	2.9 (0.90)	Partially acceptable (56.3%)	1.2 (0.60)	0.1 (0.51)	48/77
Case 22	2.0 (1.2)	3.3 (1.0)	Fully acceptable (77.1%)	1.7 (0.48)	0.6 (0.76)	48/77
Case 23	3.5 (1.0)	2.5 (0.65)	Partially acceptable (62.5%)	1.1 (0.60)	−0.3 (0.49)	48/77
Case 24^*^	5.0 (0.25)	2.6 (0.73)	Partially acceptable (52.3%)	1.0 (0.69)^*^		47/77
Case 25	2.0 (1.1)	1.2 (0.58)	Fully acceptable (56.5%)	1.4 (0.65)	−0.4 (0.65)	46/77
Case 26	3.8 (0.51)	2.3 (0.63)	Partially acceptable (53.3%)	1.3 (0.60)	−0.5 (0.36)	45/77
Case 27	2.3 (0.86)	2.2 (0.68)	Partially acceptable (73.3%)	0.96 (0.52)	−0.0 (0.51)	45/77
Case 28	3.0 (0.29)	2.7 (0.45)	Fully acceptable (66.6%)	1.6 (0.52)	−0.1 (0.21)	45/77
Case 29	3.1 (0.77)	2.6 (0.54)	Partially acceptable (51.1%)	1.2 (0.67)	−0.2 (0.36)	45/77
Case 30	4.1 (1.2)	2.5 (0.63)	Unacceptable (46.7%)	0.62 (0.65)	−0.4 (0.55)	45/77
Case 31	4.2 (1.2)	2.4 (0.62)	Partially acceptable (57.8%)	1.0 (0.65)	−0.5 (0.54)	45/77
Case 32	3.0 (0.21)	2.6 (1.1)	Fully acceptable (91.1%)	1.9 (0.28)	−0.2 (0.36)	45/77

* *Cases with the poorest agreement between participants (see also Supplementary Figure 1)*.

### Identifying and Eliminating Questions With the Poorest Agreement

In the first step, we identified cases for which the agreement among respondents was the poorest for complication grading, for surgical indication grading, and for subjective rating. Therefore, we eliminated cases showing the greatest spread among respondents, that is, the greatest standard deviation (see *Materials and Methods*). These were cases 6 and 7 for complications, 7 and 12 for surgical indication, and 6 and 24 for subjective rating. These cases were deleted and not considered for further statistical analyses.

Supplementary Figure 1 shows, for complications, the relative frequency of complication grading for cases 6 and 7 with poor agreement (high standard deviation). Cases 5 and 8 are taken as examples of cases with better agreement (low standard deviation) to illustrate the concept. A similar approach was used for surgical indication and subjective rating.

### The Indication to Complication Index

We then created the ICI by dividing the grading of indication by the grading of complication. We took the log of these values, as homoscedasticity assumption was not met for the ICI. Mean scores and standard deviations for each clinical case are shown in Table 3.

### Performance of the Indication to Complication Index

To better understand the ICI and how it could be used as a tool to quantify a complication with respect to the indication, we performed subgroup analyses. For all cases rated to be unacceptable (cases 14, 20, and 30), the ICI was below 0 (mean −0.17, *SD* 0.054). For cases deemed to be acceptable with a large consensus (>two thirds of respondents) (cases 3, 9, 10, 15, 16, 18, 22, 28, and 32), the ICI was above 0 (mean 0.075, *SD* 0.17). For the other cases, the mean ICI was −0.036 (*SD* 0.13).

### Correlation With Subjective Rating

**Figure 1:**
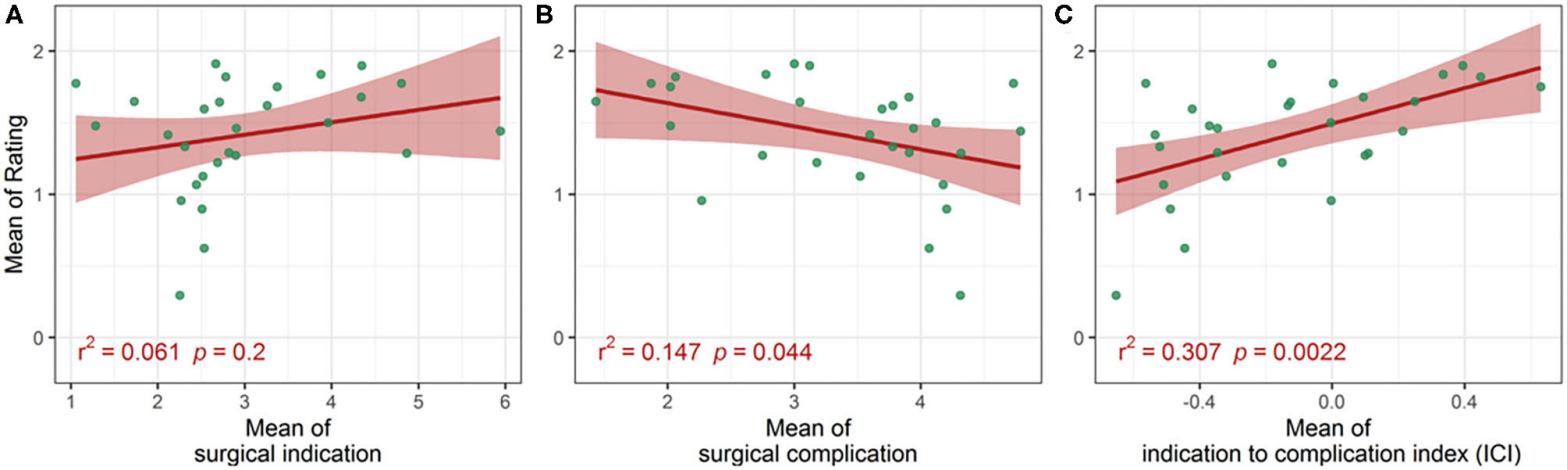
Correlation between the mean (aggregated over each case) of the subjective rating and the mean of the surgical indication **(A)**; the mean of the surgical complication **(B)**; and the mean of the Indication to Complication Index (ICI; **C**).

When looking at the correlations between the three indexes (surgical complication, surgical indication, and ICI) and the mean of subjective rating, both surgical complication and ICI were significantly correlated to the subjective rating, while surgical indication failed to reach statistical significance at the 5% level (Figure 1). Surgical complication and the mean of subjective rating showed a weak correlation (Pearson correlation *r*^2^ = 0.147, *p* = 0.044), while the correlation between the mean ICI to the mean of subjective rating was strong (Pearson correlation *r*^2^ = 0.307, *p* = 0.0022).

**Figure 2:**
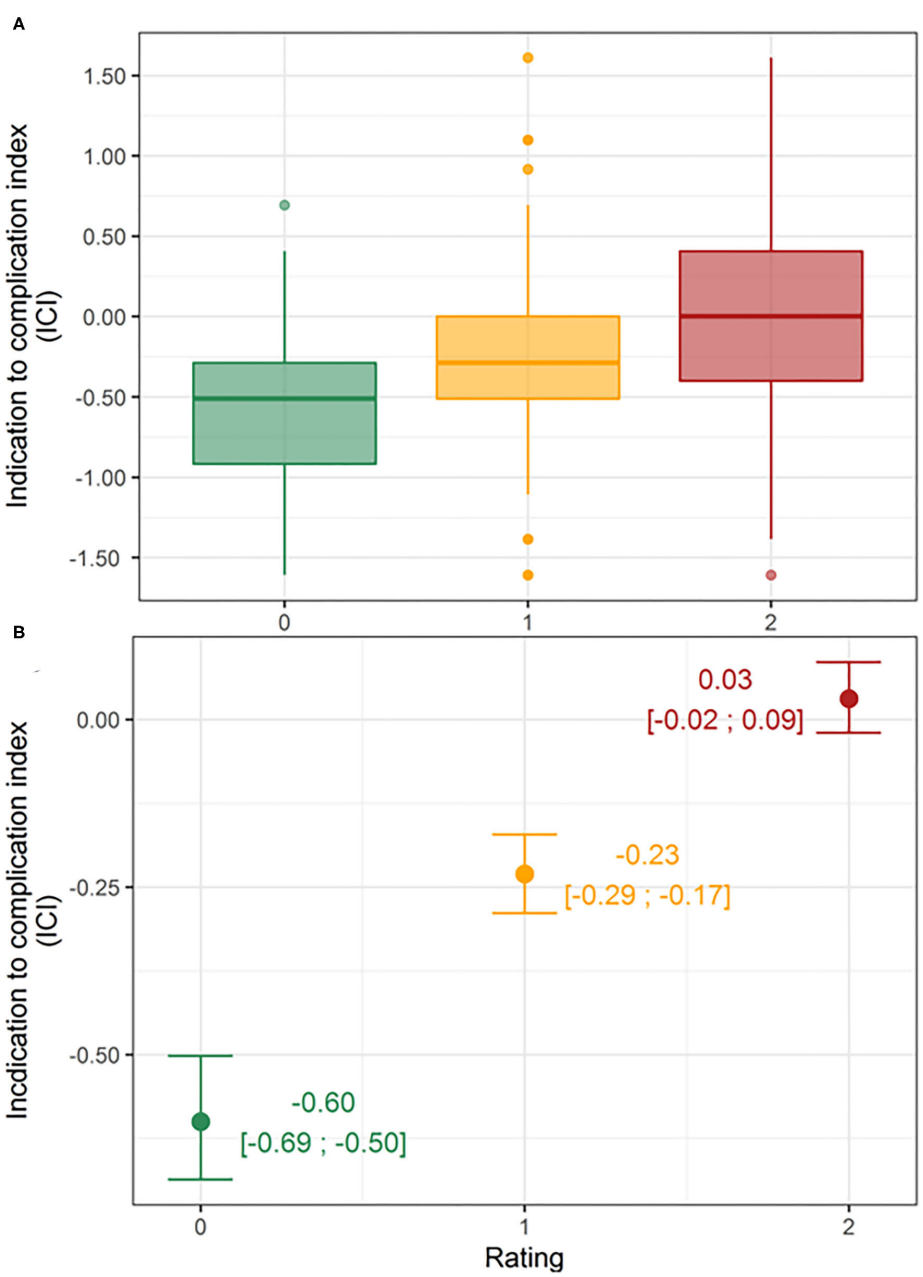
Distributions of Indication to Complication Index (ICI) by rating group. Boxplots **(A)** and estimated means (95% CI) per rating group derived by the linear mixed-effects model **(B)**.

When comparing the distribution among groups with the linear mixed-effects model, statistical analysis revealed a significant difference between the three groups (*p* < 0.001) (Figure 2). The estimated means [95% confidence interval (CI)] of the ICI were −0.6 (95% CI: −0.69 to −0.50) for subjective rating 0 (unacceptable), −0.23 (95% CI: −0.29 to −0.17) for subjective rating 1 (partially acceptable), and 0.03 (95% CI: −0.02 to 0.09) for subjective rating 2 (fully acceptable) (Figure 2). The residual plot is available as a Supplementary Figure 2.

## Discussion

Accuracy and transparency in reporting surgical complications are expected in modern clinical care. It is also needed to guarantee and report quality control. The latter is increasingly required by health care policy makers of most national health systems. The development of a common language of complication grading such as the Clavien–Dindo has helped in this regard.

Reporting surgical complications should ultimately allow to avoid them and reduce their number (11). Reporting the extent by which they were corrected is a very good step in that regard. We felt that, especially in the field of otolaryngology–head and neck surgery, one has however to consider why the patient was operated on primarily to be able to better understand the circumstances and the clinical setting of the complication.

We showed that the grade of surgical complication and the subjective rating of the respondents inversely correlated. Intuitively, most would agree that the more serious the complication, the less acceptable it is, making this result logical. However, when grading the surgical complication with respect to the surgical indication using the ICI, the correlation with the subjective rating increased dramatically. This confirms our initial hypothesis that most respondents will judge a complication as acceptable or not according to its grade but keeping in mind the surgical indication.

Another concept that we felt necessary to also integrate in our system was how reversible a complication is. A reversible complication may be bothersome but everything will eventually be back to normal. A complication leading to permanent loss of function means a sequela for the patient (12). Patients perceive such surgical complications more severely (13). Therefore, it seems particularly important especially in the field of otolaryngology–head and neck surgery that treats and cures diseases around sensory organs and cranial nerves (14).

Our index, although reflecting better the subjective rating of a complication, does not rely on a surgeon's intuition. Some authors have advocated the use of a visual analog scale (VAS) to grade complications (15), which is certainly too vague as a tool to rely on (16). Other commonly used systems for grading surgical complications are the Accordion Severity Grading System, the Memorial Sloan–Kettering Cancer Center (MSKCC) System, or the T92 system (17). Although they differ somewhat on the grading system or the relative weighting of sequelae, all of them have a similar approach to the Clavien–Dindo classification system. The index proposed in this study is a novel approach and has many potential advantages, as discussed above.

Limitations of this study are the fact that, in order to calculate the index, we had to transform ordinal numeric variables into a scale. This needs to be borne in mind when interpreting our results as a complication grade 6 on a scale is twice “as bad” as complication grade 3, although no such assumption can be made when using ordinal numeric variables (grade 6 just being three categories worse than grade 3) (10).

Further, questions with poor agreement with respective high variations among respondents had to be removed. Although it remains hypothetical, the phrasing of the question may have been somewhat imprecise and some questions provided more details about a particular case than others (e.g., case 6, no mention of what was done against permanent sensorineural hearing loss). We sought for brevity in the phrasing of the clinical scenarios not to make the questionnaire too lengthy for the respondents. This may have led to some difficulty for respondents to get an accurate picture of the presented scenario. The phrasing of each scenario shall be refined to provide a concise, yet clear picture of each case with accurate description of the surgical indication, complication, and what was done to resolve it.

Only 68.4% of respondents completed the survey fully, substantiating the fact that the survey or the questions may have still been too long (see Table 3, last column). The survey was sent out in English language only, which might have led to some difficulty for non-native English speakers. However, the case presentation was kept simple and the questionnaire was intentionally designed to be well-understandable for international participants with a basic knowledge of the (scientific) English language that was assumed in all addressed individuals to be present. All questions have been reviewed by the entire study team for consistency and readability and to ensure reproducible answers. The validity of the survey is somewhat limited, as we do not know the percentage of surveyed colleagues who actually filled out the survey, since multiple e-mails were sent out and forwarded, however without tracking of how many people were asked to fill out the survey.

It may also have been difficult to determine if some complications are to be graded as loss of organ function (which would require a grading of 5) (case 7: case with spinal accessory nerve paresis: is this really a “loss of organ function”?). Our data rely only on theoretical, albeit real cases-based, scenarios. It gives the opportunity for further studies with prospective and real-life validation of the index.

When correctly applied, it may also serve as a fair tool to compare complications among institutions and colleagues (18). Since the disease spectrum and severity may vary widely among them, it will be very useful to be able to compare complications with respect to the surgical indication. The original idea of the study emerged after a mortality and morbidity session, in which we felt that there was no proper way of assessing complications with respect to the surgical indication. It does not however encompass whether the complication was due to poor preoperative planning, surgical technique, or postoperative management and if the complication could have been prevented or not. Including such points would possibly enhance the grading of the acceptability and make it more transparent. Any classification system needs however to be simple enough to remain applicable in daily clinical practice.

In general, our index can be applied to different complications within one subject resulting in different indices, showing which one of them would be acceptable or not. This is a clear strength of our classification, as it is not limited to the most severe complication compared to other reporting systems.

In conclusion, we describe a new index for grading of surgical complications with respect to the surgical indication. Based on the Clavien–Dindo classification, it allows an appraisal of a complication according to the circumstances of the operation.

## Data Availability Statement

The raw data supporting the conclusions of this article will be made available by the authors, without undue reservation.

## Author Contributions

GM and MS: basic study idea. MS: surveying. GM: manuscript drafting. NA and GM: statistics and figures. All authors participated to the design, drafting of the scenarios, participated substantially to the study, and approved the final version of the manuscript.

## Conflict of Interest

The authors declare that the research was conducted in the absence of any commercial or financial relationships that could be construed as a potential conflict of interest.

## Supplementary Material

Supplementary Figure 1 Relative frequencies of complication grading for cases 5, 6, 7, and 8. Cases 5 and 8 show good agreement with homogeneous distribution of gradings; meanwhile, cases 6 and 7 had a rather heterogeneous distribution of gradings.

Supplementary Figure 2 Residual plot for linear mixed-effects model.
