# Supplementary material for: Assessment of Surgical Complications With Respect to the Surgical Indication: Proposal for a Novel Index
Source: Front Surg. 2021 Feb 18;8:638057. doi: 10.3389/fsurg.2021.638057 (PMC7930554; doi:10.3389/fsurg.2021.638057)
Supplement: Supplementary file 1 [file Data_Sheet_1.docx]

**Supplemental Material 1 : Clinical scenarios for survey**

**Case 1** Septoplasty patient for nasal breathing impairment gets a symptomatic septal perforation needing surgery

**Case 2** Septoplasty patient gets asymptomatic septal perforation

**Case 3** 42yo patient with subtotal glossectomy, bilateral neck dissection and free flap reconstruction for T4a N2b border of tongue cancer requiring flap revision for anastomosis insufficiency at d2 and total of 10 days of intensive care unit for delirium.

**Case 4** 70yo patient with partial glossectomy and sentinel lymph node biopsy for T1 N0 left border of tongue cancer with two episodes of postoperative bleeding from lingual artery and temporary tracheostomy.

**Case 5** 40yo patient with deep parotid lobe lipoma. Normal postoperative facial nerve function however wound hematoma requiring revision on day 2

**Case 6** 45yo patient with tympanoplasty for chronic otitis with central perforation. Postoperatively permanent severe sensorineural hearing loss

**Case 7** 49yo patient with p16 positive- T2 N1 carcinoma of right tonsil. Undergoes tumor tonsillectomy and neck dissection. Remains free of disease at 2 years follow-up however suffers disabling paresis of spinal accessory nerve (requires change of job)

**Case 8** 40yo patient with chronic rhinosinusitis with nasal polyps undergoes sphenoethmoidectomy with intraoperative CSF leak without meningitis but requiring revision in general anesthesia

**Case 9** Adenocarcinoma patient with endoscopic transnasal tumor debulking gets CSF leak requiring revision in general anesthesia

**Case 10** Aesthetic rhinoplasty patient gets an infection that can be solved with antibiotics

**Case 11** Nasal reconstruction suffers a flap necrosis requiring revision in general anesthesia

**Case 12** Surgery for refractory epistaxis (not acutely bleeding) gets septicemia needing intensive care unit treatment

**Case 13** Spontaneous carotid aneurysm bleeding (epistaxis patient) in sphenoid is managed transnasally but gets blind.

**Case 14** 23yo patient with deafness and tinnitus after tympanoplasty to close central perforation.

**Case 15** 35yo patient with dizziness after cochlear implantation.

**Case 16** 8yo patient with unexpected conductive hearing loss after surgical removal of a cholesteatoma

**Case 17** 44yo patient with permanent facial paralysis after resection of a large vestibular schwannoma.

**Case 18** 44yo patient with CSF leak after removal of a large vestibular schwannoma, resolving with lumbar drain after 5 days

**Case 19** 45yo Patient suffering from large vestibular schwannoma (with compression of brainstem). Postoperative facial palsy (initially House Brackman grade V improving to grade II over 18 months)

**Case 20** Otosclerosis patient loses hearing permanently on one side after stapedotomy of the second side

**Case 21** 67yo Patient suffering with small vestibular Schwannoma. Postoperative meningitis leading to prolonged (non-intensive care unit) hospitalization and antibiotic treatment.

**Case 22** Cholesteatoma patient presents with dizziness and acute transient hearing loss after surgery.

**Case 23** Patient with recurrent otitis externa due to hyperostosis develops fibrous atresia with conductive hearing loss postoperatively.

**Case 24** 58yo patient with injection laryngoplasty (medialization) for unilateral vocal fold immobility with glottal insufficiency gets severe shortness of breath needing intensive care unit treatment

**Case 25** 27yo transgender patient (MtF) with prominent thyroid cartilage (Adam’s apple) for chondrolaryngoplasty (thyroid cartilage reduction) gets postoperatively mild voice weakness

**Case 26** 54yo patient with prominent Reinke’s edema for microsurgical reduction develops after surgery a clinical significant synechia in the anterior glottal commissure needing revision surgery in general anesthesia

**Case 27** 89yo patient with Botulinum toxin type A injection into the thyroarytenoid muscle for spasmodic dysphonia develops severe dysphagia with aspiration requiring iv antibiotics.

**Case 28** Sialendoscopy of the submandibular gland for stone removal needs revision surgery in local anesthesia due to scaring of the papilla region.

**Case 29** Intratympanic steroid administration for persisting sensorineural hearing loss after sudden deafness ends up in a persisting tympanic membrane perforation after three injections.

**Case 30** Sialendoscopy (recurrent swelling due to lithiasis) with combined approach in the posterior floor of mouth leads to a permanent injury of the lingual nerve.

**Case 31** 45yo male cook losing sense of taste (must change job) on the ipsilateral side of the tongue after stapes surgery for otosclerosis.

**Case 32** 19yo patient bleeds 7 days after tonsillectomy which can be handled in local anesthesia
