# Supplementary figures and images for: Assessment of Surgical Complications With Respect to the Surgical Indication: Proposal for a Novel Index
Source: Front Surg. 2021 Feb 18;8:638057. doi: 10.3389/fsurg.2021.638057 (PMC7930554; doi:10.3389/fsurg.2021.638057)

Percent of answers

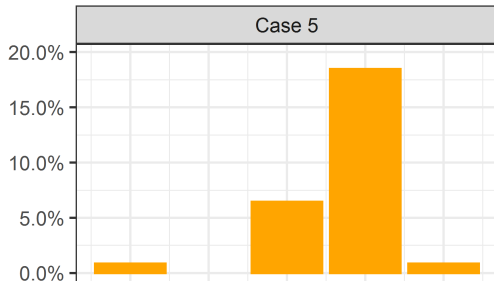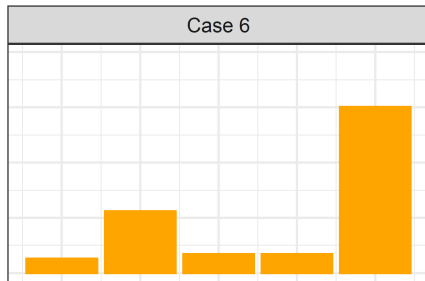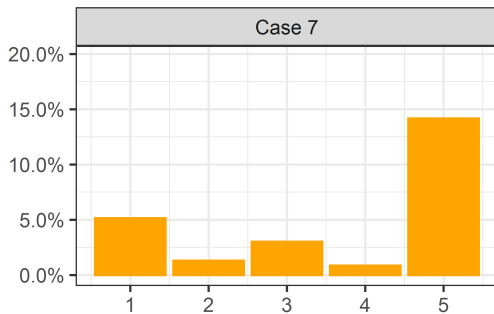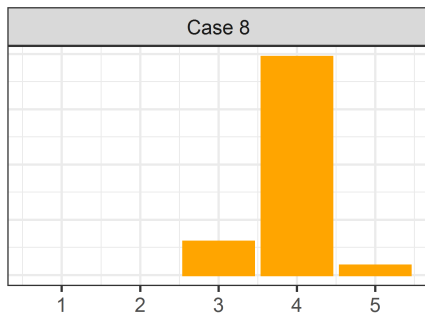

Surgical complication grading

Supplement: Supplementary Figure 1 — Relative frequencies of complication grading for cases 5, 6, 7, and 8. Cases 5 and 8 show good agreement with homogeneous distribution of gradings; meanwhile, cases 6 and 7 had a rather heterogeneous distribution of gradings. [file Image_1.pdf]

resid(., type = "pearson")

1  
0  
-1

-0.8

-0.6

-0.4

-0.2

0.0

0.2

fitted(.)

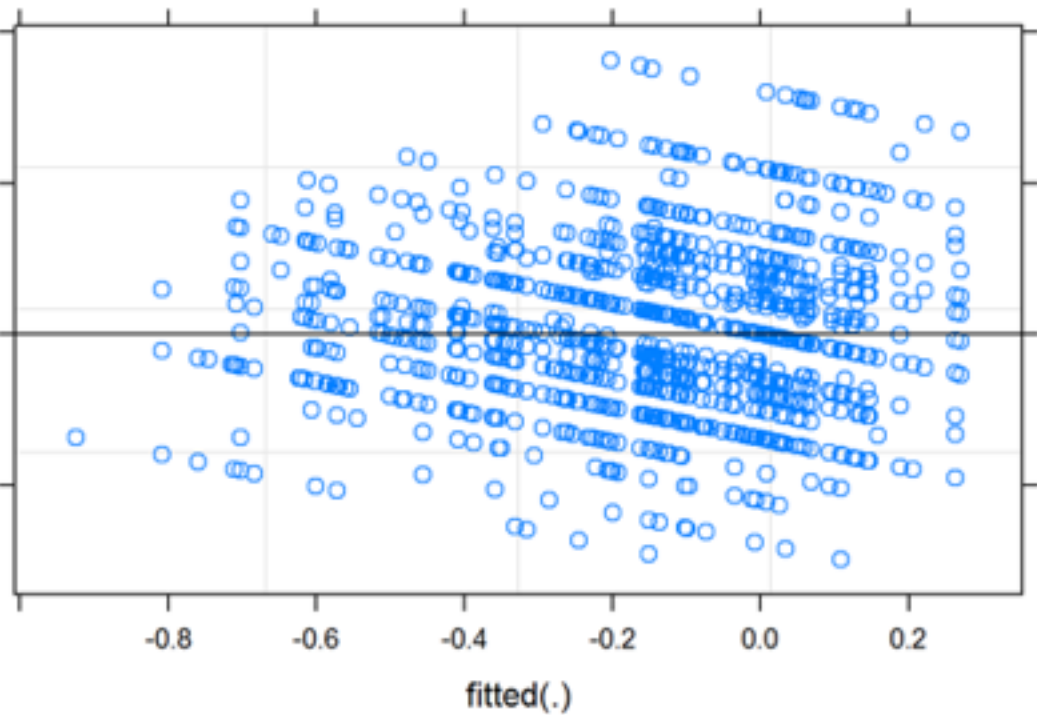

Supplement: Supplementary Figure 2 — Residual plot for linear mixed-effects model. [file Image_2.pdf]
